# Exploring Vascular Contributions to Migraine: Association Analysis of Small Vessel Disease Genetic Variants

**DOI:** 10.3390/genes17050541

**Published:** 2026-05-01

**Authors:** Zizi Molaee, Mohammed Al-Fayyadh, Robert A. Smith, Neven Maksemous, Lyn R. Griffiths

**Affiliations:** Genomics Research Centre, School of Biomedical Sciences, Faculty of Health, Queensland University of Technology, 60 Musk Ave, Kelvin Grove, QLD 4059, Australia; zizi.molaee@hdr.qut.edu.au (Z.M.); mohammed.alfayyadh@hdr.qut.edu.au (M.A.-F.); r157.smith@qut.edu.au (R.A.S.); n.maksemous@qut.edu.au (N.M.)

**Keywords:** migraine, cerebral small vessel disease, CTC1, ASTN2, vascular genetics, telomere biology, association analysis

## Abstract

**Background**: Migraine is a complex neurovascular disorder with a substantial genetic component, yet many contributing loci remain poorly characterised. **Methods**: This study investigated the association between 21 biologically prioritised single nucleotide variants (SNVs) and migraine susceptibility in a case-control cohort of 548 individuals of European ancestry, of whom 304 (164 cases, 140 controls) remained after quality control and principal component analysis (PCA). Genotyping was performed using a targeted Sequenom MassARRAY platform, and substantial missingness (mean 30.3% per SNV) was addressed using multiple imputation by chained equations (MICE). Association testing was conducted using three complementary logistic regression frameworks: unadjusted single-variant analysis, covariate-adjusted marginal models, and a multivariable joint model incorporating all SNVs with L2 regularisation. **Results**: Across analyses, two variants in *ASTN2* (rs1052053 and rs6478241) showed the most robust associations with migraine, surviving Bonferroni correction in the joint model (*p* = 0.001 and *p* = 0.002, respectively) and false discovery rate (FDR) correction in marginal models (q = 0.003 for both). A third variant, rs7304841 (12p12), demonstrated a risk-increasing effect that reached FDR significance in marginal analysis (q = 0.035) and remained nominally significant in the joint model. In contrast, rs62624978 in *CTC1* showed a strong signal in unadjusted analysis (OR = 0.217, *p* = 0.0014) and remained nominally significant after adjustment (*p* = 0.011), although it did not survive multiple-testing correction in imputed models. The joint model demonstrated good discriminatory performance (AUC = 0.822), though this is not intended as a predictive tool. Biologically, implicated loci suggest contributions from both neuronal circuit organisation (*ASTN2*) and telomere and vascular maintenance pathways (*CTC1*), supporting a broader neurovascular model of migraine susceptibility. **Conclusions**: These findings are consistent with shared genetic architecture between migraine and microvascular dysfunction, potentially involving endothelial integrity, neurovascular coupling, and cortical excitability mechanisms.

## 1. Introduction

Migraine is a common neurological disorder affecting about 15% of the global population, characterised by recurrent headaches and symptoms like nausea, photophobia, and phonophobia [1,2]. Despite its considerable socio-economic impact, the pathophysiological mechanisms underlying migraine remain incompletely understood [3]. Emerging evidence suggests that vascular factors and genetic components play significant roles in migraine pathogenesis [4]. Cerebral small vessel disease (SVD) affects the small arteries, arterioles, capillaries, and venules of the brain [5]. It is a leading cause of stroke and cognitive decline, commonly identified through neuroimaging markers such as white matter hyperintensities, lacunar infarcts, and microbleeds [5,6]. Individuals with migraine, especially those experiencing aura, have a higher prevalence of these neuroimaging markers, indicating a potential link between migraine and SVD [7,8]. Both migraine and cerebral small vessel disease (SVD) have genetic components [9,10,11]. Genome-wide association studies have identified genetic variants linked to migraine susceptibility that involve vascular functions, endothelial integrity, and cerebral blood flow regulation [12,13,14]. Similarly, variants associated with SVD affect vascular remodelling, blood–brain barrier function, and inflammatory responses in cerebral vessels [15,16]. This genetic overlap suggests shared molecular pathways contributing to both conditions [17,18]. Cortical spreading depression (CSD), a wave of neuronal and glial depolarisation across the cerebral cortex, is central to migraine pathophysiology [19]. It can trigger the aura and activate pathways leading to headache pain [20]. Alterations in cerebral microvasculature seen in SVD may facilitate CSD by affecting cerebral autoregulation and endothelial function, suggesting a mechanistic link between SVD and migraine [21].

Despite these converging lines of evidence, the contribution of SVD-associated genetic variants to migraine susceptibility remains insufficiently characterised. In this study, we performed a targeted association analysis of 37 SVD-related single-nucleotide variants (SNVs) in a well-defined migraine case–control cohort. By interrogating variants identified through familial migraine analyses, previously reported variants from our group’s studies on hemiplegic migraine and published vascular genetics literature, we aimed to evaluate whether SVD-linked genetic loci may contribute to migraine susceptibility and to further explore the vascular genetic architecture underlying migraine.

## 2. Methods

### 2.1. Study Population

A total of 548 unrelated Caucasian individuals were initially recruited for this study, comprising 267 migraine cases and 281 neurologically unaffected controls. Participants were enrolled through the Genomics Research Centre (GRC) at Queensland University of Technology (QUT), Brisbane, Australia. Migraine diagnoses were established by experienced clinicians according to the International Classification of Headache Disorders (ICHD) criteria [22]. Control individuals were selected on the basis of the absence of any personal or family history of migraine or other significant neurological disorders. Written informed consent was obtained from all participants prior to enrolment, and peripheral blood samples were collected for genomic DNA extraction using the standard salting-out protocol [23].

Following quality control (QC) filtering and integration of genotype, phenotype, and principal component analysis (PCA) data, 304 individuals (164 cases, 140 controls) were retained for association analyses after merging with PCA data (see Section 2.4). Covariates available for all retained individuals included age, sex, and the top four principal components (PC1–PC4).

### 2.2. SNV Selection and Genotyping

Single nucleotide variants (SNVs) selection followed a biologically informed candidate variant strategy. SNVs were prioritised from three complementary sources: (i) rare variants identified through whole-exome sequencing (WES) in multigenerational migraine families showing evidence of segregation with disease; (ii) variants previously reported by Molaee et al. (2025) in a hemiplegic migraine cohort [24]; and (iii) variants reported in the literature to be associated with cerebral small vessel disease (SVD) or vascular-related pathways. Priority was given to missense variants and loci with putative functional relevance in endothelial biology, telomere maintenance, and neurovascular signalling. All variants were pre-selected before conducting the association analysis.

A total of 37 SNVs were selected for targeted genotyping. Genotyping was performed using a custom Sequenom MassARRAY platform (Agena Bioscience, San Diego, CA, USA). Assays were designed using AgenaCx software v1.4 according to manufacturer specifications [25]. Genomic DNA concentrations were standardised to 20 ng/µL prior to polymerase chain reaction (PCR) amplification. Post-PCR processing included shrimp alkaline phosphatase (SAP) treatment, iPLEX Gold single-base extension, desalting, and transfer onto SpectroCHIPs using the Nanodispenser RS1000 (San Diego, CA, USA). Genotypes were resolved via matrix-assisted laser desorption/ionisation time-of-flight (MALDI-TOF) mass spectrometry and assigned using SpectroTYPER v4. Samples with missing calls were re-analysed by High Resolution Melt (HRM) analysis on a QIAGEN Rotor-Gene Q platform using SYTO-9 dye chemistry (QIAGEN, Hilden, Germany).

### 2.3. Quality Control and Data Processing

Standard QC procedures were applied using PLINK v1.90 [26,27]. Variants showing significant deviation from Hardy–Weinberg equilibrium (HWE) in controls were excluded (14 SNVs removed). Relatedness analysis confirmed all individuals were unrelated. Relaxed missingness thresholds (≤10%) were applied to maximise data retention, given the modest cohort size [28]. No minor allele frequency (MAF) threshold was imposed, consistent with rare-variant candidate analyses [29]. Linkage disequilibrium (LD) pruning (--indep-pairwise 50 10 0.2) was applied exclusively for PCA computation; two additional SNVs were removed. The full unfiltered variant set was retained for association analyses. Following all QC steps, 21 SNVs were carried forward.

### 2.4. Population Stratification

**Figure 1:**
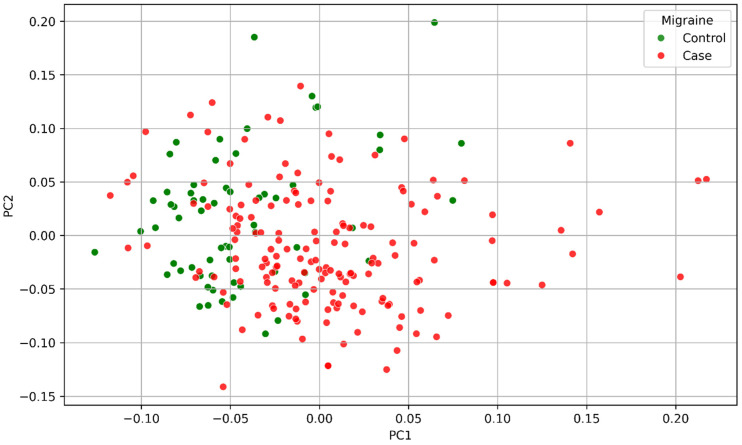
Principal component analysis of the study sample (*n* = 304 after exclusion of samples with a call rate < 50%). PC1 is plotted against PC2 coloured by case–control status. Cases (red) and controls (green) show broad interleaving with no systematic separation, consistent with a genetically homogeneous Caucasian sample. PC1–PC4 were included as covariates in all logistic regression models.

PCA was performed on genome-wide genotype data using PLINK v1.90--pca [27]. Prior to PCA, samples with a genotyping call rate below 50% were excluded (--mind 0.5), as individuals with excessive missing data preclude computation of the genetic relationship matrix (GRM). This resulted in a PCA sample of 304 individuals. The top 20 principal components were extracted; the top four (PC1–PC4) were included as covariates in all logistic regression models. The selection of four components is consistent with standard practice in genetic association studies of homogeneous-ancestry samples. PCA results were visualised by plotting PC1 against PC2, coloured by case–control status (Figure 1).

### 2.5. Missing Data Characterisation and Imputation

Genotype missingness was characterised prior to analysis. Per-SNV missing rates were computed across all 304 participants retained after PCA merging (Supplementary Figure S1). Missing rates ranged from 0.0% (rs1008539) to 87.5% (rs880315), with a mean of 30.3%. Eleven of 21 SNVs exceeded the 20% threshold. Complete-case analysis retaining only samples with fully observed genotypes across all 21 SNVs was not feasible, as only five participants met this criterion (<2% of the analytic sample).

To formally assess the missing data mechanism, logistic regression was used to test whether the probability of missingness at each SNV was associated with case–control status after adjustment for age, sex, and PC1–PC4 [30]. Missingness was significantly associated with case–control status in 13 of 21 SNVs (*p* < 0.05; Supplementary Table S1), indicating that data were missing at random (MAR) rather than missing completely at random (MCAR), and motivating the use of multiple imputation.

Missing genotypes were imputed using multiple imputation by chained equations [31] via scikit-learn’s IterativeImputer [32]. *M* = 20 imputed datasets were generated, matching the mean missingness rate [33]. The imputation model included all 21 SNVs, age, sex, PC1–PC4, and the outcome variable as predictors. Imputed dosage values were constrained to [0, 2] and rounded to the nearest integer.

### 2.6. Statistical Analysis

Three complementary logistic regression analyses were performed. First, per-SNV marginal models were fitted using PLINK v1.90 on the unadjusted dataset (*n* = 548). Second, per-SNV marginal models were fitted in Python 3.12.0 on the MICE-imputed data (*n* = 304), with each SNV adjusted independently for age, sex, and PC1–PC4. Third, a multivariable joint model was constructed, including all 21 SNVs simultaneously with the same covariates. Ridge penalisation (L2 regularisation, C = 1.0) was applied to the joint model to stabilise estimates.

For Python-based models, logistic regression was fitted on each of the *M* = 20 imputed datasets independently, and results were pooled using Rubin’s rules [34]: point estimates were averaged, and total variance was computed as the sum of within-imputation and between-imputation variance, the latter scaled by (1 + 1/M). Pooled odds ratios (ORs), 95% confidence intervals (CIs), and *p*-values were derived using a standard normal approximation.

Nominal significance was defined at *p* < 0.05. Bonferroni correction was applied across 21 SNVs (α = 0.05/21 = 2.38 × 10^−3^). False discovery rate (FDR) correction was applied using the Benjamini–Hochberg (BH) procedure (q < 0.05) [35]. As a sensitivity analysis, complete-case ORs were compared directionally against MICE-imputed ORs (Supplementary Figure S2).

## 3. Results

### 3.1. Population Stratification

PCA of genome-wide genotype data from 304 individuals (after exclusion of samples with genotyping call rate < 50%) revealed no evidence of gross population stratification. Cases and controls showed broad overlap across the principal component space, with no systematic separation along PC1 or PC2 (Figure 1). A small number of individuals with extreme PC values were visible at the periphery but did not cluster distinctly and were retained. These findings are consistent with a genetically homogeneous Caucasian cohort and support the use of PC1–PC4 as covariates to account for residual ancestry-related confounding.

### 3.2. Genotype Missingness

Per-SNV missingness rates ranged from 0.0% (rs1008539) to 87.5% (rs880315), with a mean of 30.3% (Supplementary Figure S1). Six SNVs had missingness below 5% (rs1008539 0.0%, rs35737219 0.7%, rs7684253 3.3%, rs1024905 3.6%, rs200735885 4.0%, rs10786156 4.6%). Four SNVs fell in the 5–20% band (rs1800127 6.2%, rs1572668 6.9%, rs7304841 7.6%, rs11624776 16.4%). The remaining 11 SNVs exceeded 20%, including six above 50%: rs1052053 (53.0%), rs140325655 (54.9%), rs34004222 (60.5%), rs8046696 (68.1%), rs62620189 (73.4%), and rs880315 (87.5%). Visual inspection of the missingness heatmap revealed structured, contiguous blocks of missing genotypes spanning both cases and controls, consistent with assay-level failure rather than participant-level dropout (Supplementary Figure S3). Formal MCAR testing identified a significant association between missingness and case–control status in 13 of 21 SNVs (*p* < 0.05), confirming a MAR mechanism and motivating MICE imputation as the primary analysis.

### 3.3. PLINK Marginal Association Analysis (n = 548, Unadjusted)

Single-variant logistic regression using PLINK v1.90 on the full 548-participant dataset identified two SNVs at nominal significance (*p* < 0.05): rs62624978 in *CTC1* (OR = 0.217, *p* = 0.0014) and rs6478241 in *ASTN2* (OR = 0.345, p = 0.0028) (Table 1). A third SNV, rs10895275 in *YAP1*, was nominally significant (OR = 0.703, *p* = 0.039). After FDR correction, rs62624978 and rs6478241 shared the lowest q-value of 0.029. After Bonferroni correction (α = 2.38 × 10^−3^), no SNV survived: the Bonferroni-corrected *p*-value for rs62624978 was 0.030, which exceeds the threshold. The remaining 18 SNVs were non-significant (*p* ≥ 0.11).

### 3.4. Joint Model, MICE-Imputed (n = 304)

**Figure 2:**
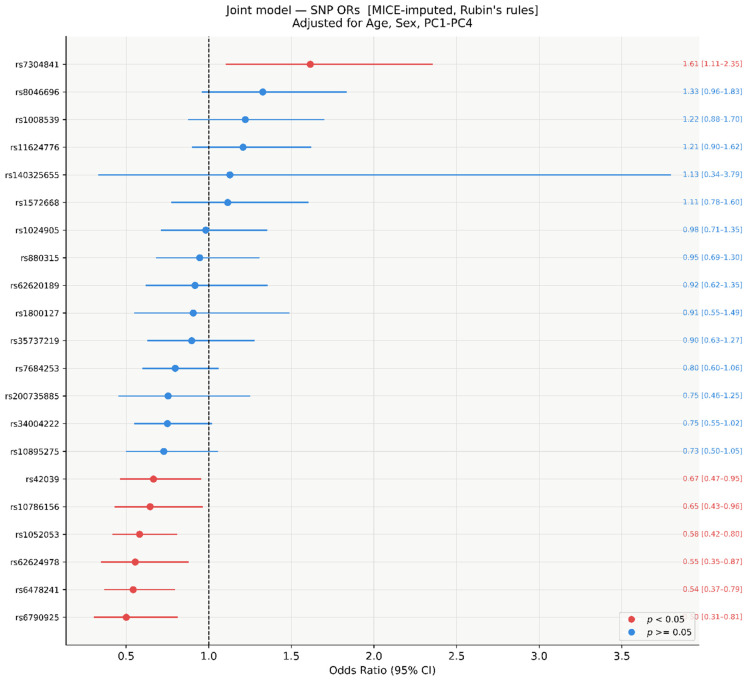
Forest plot of SNV ORs from the joint logistic regression model (MICE-imputed, *n* = 304). All 21 SNVs fitted simultaneously, adjusted for age, sex, and PC1–PC4. Red: *p* < 0.05; blue: *p* ≥ 0.05. Results pooled across *M* = 20 imputed datasets using Rubin’s rules.

**Figure 3:**
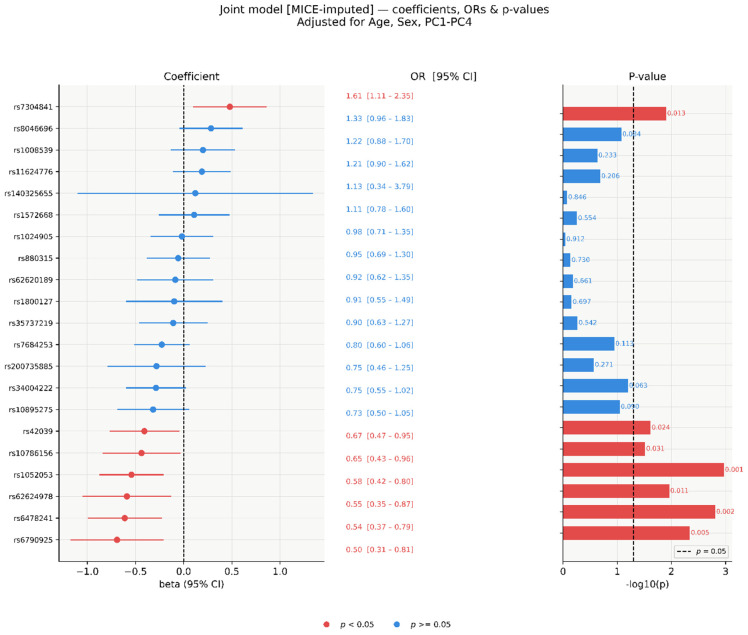
Joint model standardised coefficients (β), ORs, and −log_10_(*p*)-values (MICE-imputed, *n* = 304). Left: β with 95% CI. Centre: OR with 95% CI. Right: −log_10_(*p*); dashed line at *p* = 0.05. Red: *p* < 0.05; blue: *p* ≥ 0.05.

In the multivariable joint model including all 21 SNVs simultaneously, fitted on MICE-imputed data (*M* = 20, *n* = 304) and pooled using Rubin’s rules, seven SNVs reached nominal significance (*p* < 0.05; Figure 2 and Figure 3). Six showed protective associations (OR < 1): rs6790925 (OR = 0.50, 95% CI 0.31–0.81, *p* = 0.005), rs6478241 (OR = 0.54, 95% CI 0.37–0.79, *p* = 0.002), rs62624978 (OR = 0.55, 95% CI 0.35–0.87, *p* = 0.011), rs1052053 (OR = 0.58, 95% CI 0.42–0.80, *p* = 0.001), rs10786156 (OR = 0.65, 95% CI 0.43–0.96, *p* = 0.031), and rs42039 (OR = 0.67, 95% CI 0.47–0.95, *p* = 0.024). One SNV showed a risk-increasing association: rs7304841 (OR = 1.61, 95% CI 1.11–2.35, *p* = 0.013). The remaining 14 SNVs were non-significant (*p* ≥ 0.063), with ORs ranging from 0.73 to 1.33. The wide CI for rs140325655 (OR = 1.13, 95% CI 0.34–3.79) reflects its 54.9% missingness rate.

**Figure 4:**
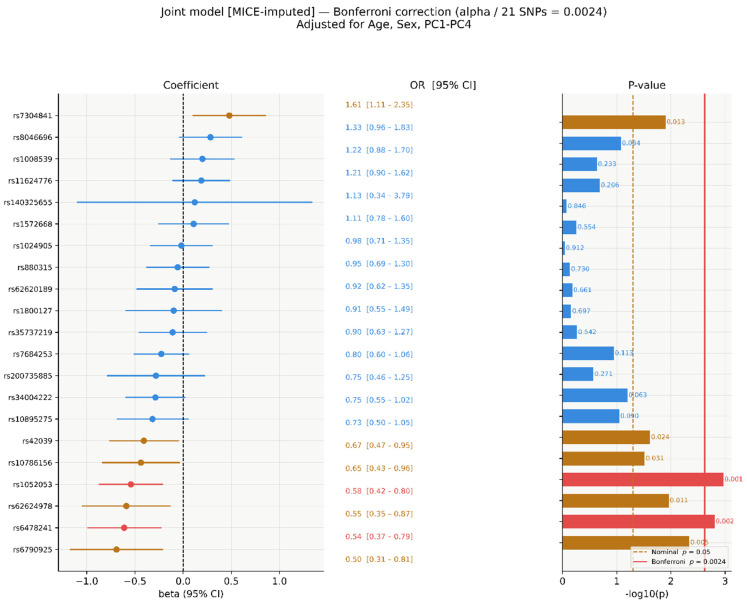
Bonferroni-corrected significance in the joint model (α/21 = 2.38 × 10^−3^). Red: Bonferroni-significant; amber: nominally significant only (*p* < 0.05); blue: non-significant. Amber dashed = nominal *p* = 0.05; red solid = Bonferroni threshold.

After Bonferroni correction, two SNVs retained significance: rs1052053 (*p* = 0.001) and rs6478241 (*p* = 0.002). Five SNVs were nominally significant only (Figure 4): rs42039 (*p* = 0.024), rs10786156 (*p* = 0.031), rs7304841 (*p* = 0.013), rs62624978 (*p* = 0.011), and rs6790925 (*p* = 0.005).

### 3.5. Per-SNV Marginal Models, MICE-Imputed (n = 304)

**Figure 5:**
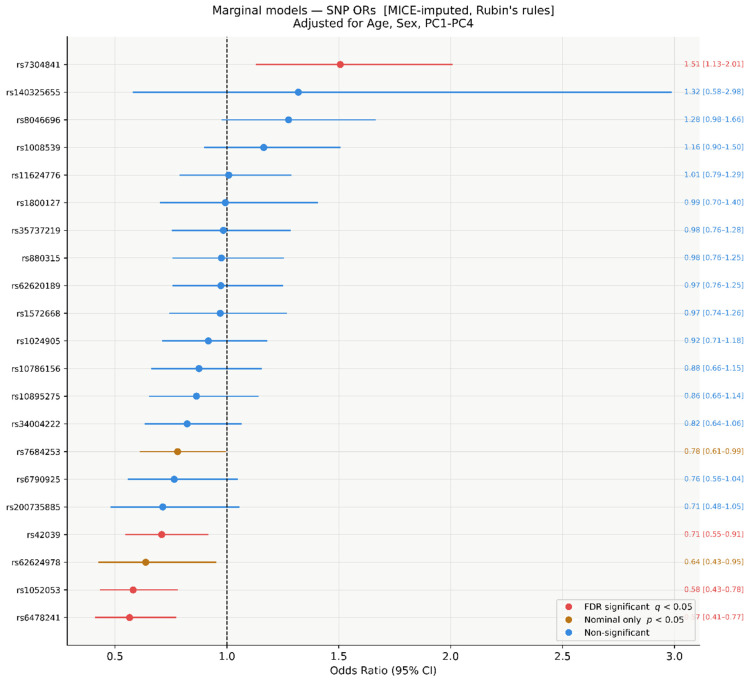
Forest plot of SNV ORs from per-SNV marginal logistic regression models (MICE-imputed, *n* = 304). Each SNV modelled independently, adjusted for age, sex, and PC1–PC4. Red: FDR q < 0.05; amber: nominal *p* < 0.05 only; blue: non-significant. Rubin’s rules pooling.

**Figure 6:**
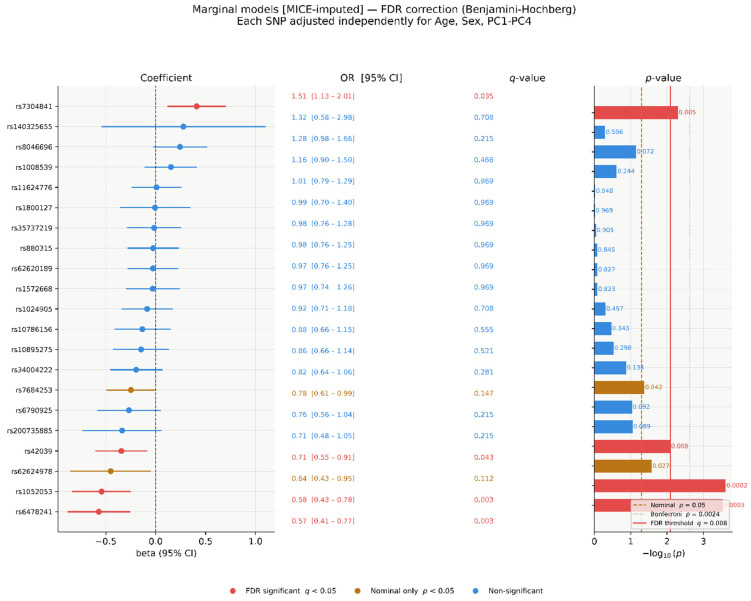
Per-SNV marginal model β coefficients, ORs, q-values, and −log_10_(*p*)-values (MICE-imputed, *n* = 304). Red solid: empirical FDR threshold (*p* = 0.008); amber dashed: nominal *p* = 0.05; grey dotted: Bonferroni *p* = 2.38 × 10^−3^.

In per-SNV marginal models on MICE-imputed data, three SNVs reached FDR significance (q < 0.05): rs7304841 (OR = 1.51, 95% CI 1.13–2.01, *p* = 0.005, q = 0.035), rs1052053 (OR = 0.58, 95% CI 0.43-0.78, *p* = 2.4 × 10^−4^, q = 0.003), and rs6478241 (OR = 0.57, 95% CI 0.41–0.77, *p* = 9 × 10^−4^, q = 0.003). The empirical FDR threshold corresponded to a raw *p*-value of 0.008. Two additional SNVs were nominally significant: rs42039 (OR = 0.71, 95% CI 0.55–0.91, *p* = 0.008, q = 0.043) and rs62624978 (OR = 0.64, 95% CI 0.43–0.95, *p* = 0.027, q = 0.112). rs7684253 was also nominally significant (*p* = 0.042, q = 0.147). The remaining 14 SNVs had q-values ≥ 0.21 (Figure 5 and Figure 6).

### 3.6. Statistical Power Analysis

A post-hoc power analysis was conducted using the Demidenko (2007) approximation for logistic regression [36], across OR = 1.5–5.0 and MAF = 0.002–0.650, at α = 0.05 (two-sided), for *n* = 548 (267 cases, 281 controls) and *n* = 304 (164 cases, 140 controls) separately, reflecting the full cohort used for PLINK analyses and the reduced post-QC sample used for MICE-imputed models, respectively. MAF values used in power calculations were obtained from the gnomAD database (non-Finnish European population), consistent with the values reported in Table 1. Power estimates reflect single-variant tests without multiple-testing correction. At *n* = 548, the study had ≥80% power to detect ORs ≥ 2.0 for variants with MAF ≥ 0.05, and ORs ≥ 2.5 for low-frequency variants (MAF ≈ 0.018). At *n* = 304, adequate power (≥80%) was retained for common variants (MAF ≥ 0.20) at ORs ≥ 2.5 but was substantially limited for rare and low-frequency variants. Detailed power estimates across the full range of MAF and OR combinations are provided in Supplementary Tables S2 and S3.

### 3.7. Model Discrimination and Classification Performance

**Figure 7:**
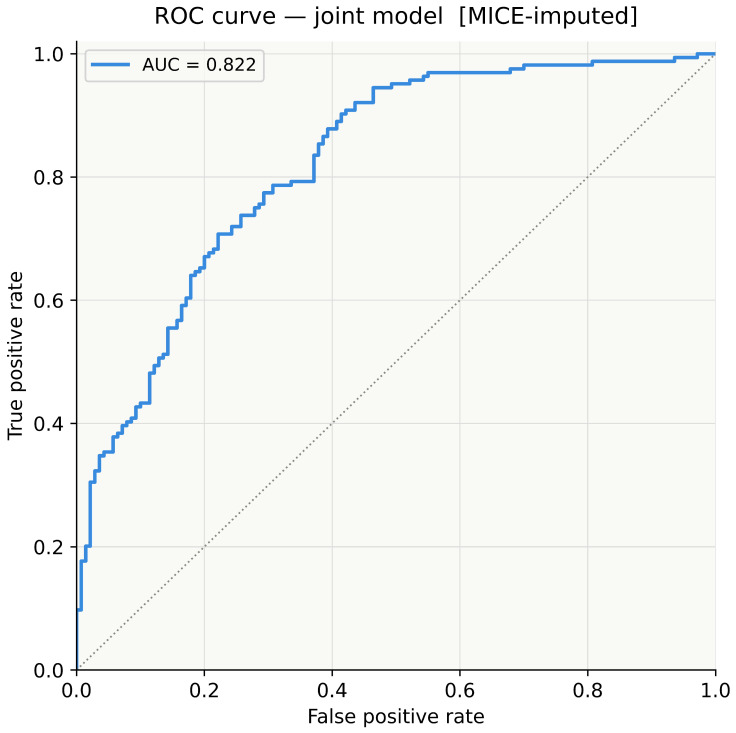
ROC curve for the joint logistic regression model (MICE-imputed, *n* = 304). AUC = 0.822. Dotted diagonal: chance-level discrimination (AUC = 0.50).

**Figure 8:**
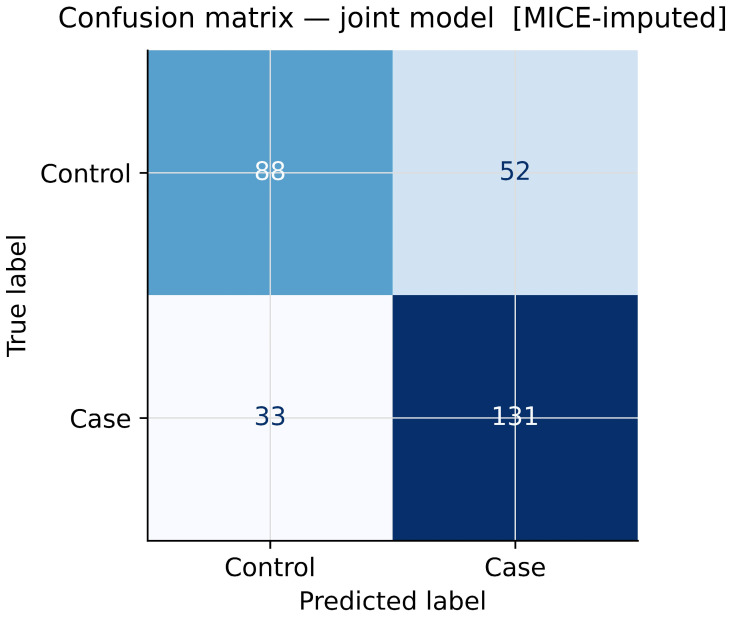
Confusion matrix for the joint model at a 0.50 classification threshold (*n* = 304). TN = 88, FP = 52, FN = 33, TP = 131. Sensitivity = 79.9%; specificity = 62.9%; overall accuracy = 72.0%.

The joint model demonstrated good discriminative ability with an area under the receiver operating characteristic (ROC) curve, the area under the curve (AUC), of 0.822 (Figure 7), substantially exceeding chance-level discrimination (AUC = 0.50). At a classification threshold of 0.50, the model correctly classified 219 of 304 participants (72.0% overall accuracy; Figure 8). Among 164 cases, 131 were correctly identified (sensitivity = 79.9%; 33 false negatives). Among 140 controls, 88 were correctly identified (specificity = 62.9%; 52 false positives). Positive predictive value was 71.6% (131/183) and negative predictive value was 72.7% (88/121).

## 4. Discussion

### 4.1. Overview of Findings

In this study, we examined the association between 21 biologically prioritised SNVs and migraine in a case–control cohort of 304 individuals retained after QC filtering and PCA merging. Three complementary analytical approaches were used: single-variant logistic regression in PLINK (*n* = 548, unadjusted), per-SNV marginal logistic regression on MICE-imputed data (*n* = 304, adjusted for age, sex, and PC1–PC4), and a multivariable joint logistic regression model including all 21 SNVs simultaneously (*n* = 304, MICE-imputed). Across these analyses, rs1052053 and rs6478241 showed the most consistent evidence of association, remaining significant after Bonferroni correction in the joint model (*p* = 0.001 and *p* = 0.002, respectively) and after FDR correction in the marginal model (q = 0.003 for both). A third SNV, rs7304841, reached FDR significance in the marginal model (q = 0.035) and remained nominally significant in the joint model (*p* = 0.013). Several other SNVs showed nominal significance across individual models but did not remain significant after correction for multiple testing.

The use of multiple imputation, covariate adjustment, and both marginal and joint modelling approaches provided a stronger framework for interpreting these findings within a neurovascular context. MICE imputation was necessary because the mean per-SNV missingness rate was 30.3%, making complete-case analysis impractical. This approach allowed retention of the full analytic sample and provided a more appropriate method for handling missing data under the observed MAR mechanism.

### 4.2. Consistency of Signals Across Analytical Frameworks

A major strength of this study is the use of three complementary analytical models, allowing the consistency of individual SNV signals to be assessed under different levels of adjustment. Differences in significance across the PLINK, marginal imputed, and joint imputed analyses are expected and should be interpreted in the context of their methodological differences. The PLINK analysis was conducted on the full cohort of 548 individuals without imputation or covariate adjustment, whereas the marginal and joint models were based on the post-QC sample of 304 individuals with both imputation and adjustment for covariates. As a result, differences between these analyses reflect differences in study design rather than contradictory findings.

In the unadjusted PLINK analysis, rs62624978 in *CTC1* showed the strongest association signal (OR = 0.217, *p* = 0.0014), while rs6478241 in *ASTN2* was also nominally significant (*p* = 0.0028). Both variants shared the lowest FDR q-value of 0.029, although neither remained significant after Bonferroni correction. In the MICE-imputed marginal model, the strongest associations were observed for rs1052053 (*p* = 2.4 × 10^−4^, q = 0.003), rs6478241 (*p* = 9 × 10^−4^, q = 0.003), and rs7304841 (*p* = 0.005, q = 0.035). In the joint model, rs1052053 and rs6478241 remained significant after Bonferroni correction, whereas rs7304841, rs6790925, rs62624978, rs42039, and rs10786156 were only nominally significant.

The reduced significance of rs62624978 in the imputed models compared with the PLINK analysis is likely related to covariate adjustment, the smaller post-QC sample size, and the low MAF of the variant (MAF = 0.018), which makes effect estimates less stable in multivariable models. In addition, 27.3% of genotypes for this SNV were missing, meaning a considerable proportion of the information was imputed rather than directly observed. Despite this, the variant remained nominally significant in the joint model (OR = 0.55, 95% CI 0.35–0.87, *p* = 0.011), and together with its strong signal in the larger unadjusted dataset, it remains a relevant finding that should be examined in independent cohorts.

Among all variants, rs1052053 and rs6478241 showed the strongest and most consistent statistical support across all models, surviving both Bonferroni correction in the joint model and FDR correction in the marginal model. rs7304841 also showed a consistent risk-increasing effect in both the marginal and joint analyses, although it did not remain significant after Bonferroni correction. These three SNVs therefore represent the strongest candidates for future replication studies.

rs6790925 in *TGFBR2* also deserves consideration. Although it was nominally significant in the joint model (OR = 0.50, 95% CI 0.31–0.81, *p* = 0.005), it was not significant in the per-SNV marginal model (*p* = 0.092, q = 0.215) or in the unadjusted PLINK analysis (*p* = 0.97). This inconsistency suggests that the joint-model signal may be influenced by collinearity with other SNVs rather than reflecting an independent association, and it should therefore not be treated as a primary finding. A similar pattern was observed for rs10895275 in YAP1, which was nominally significant in the unadjusted PLINK analysis (OR = 0.703, *p* = 0.039) but became non-significant in both the MICE-imputed marginal (*p* = 0.298) and joint (*p* = 0.090) models, indicating that the initial signal was not maintained after adjustment for covariates and missing data.

### 4.3. Biological Interpretation: CTC1 and Telomere Maintenance

The association of rs62624978 with migraine susceptibility is biologically noteworthy. *CTC1* encodes a key component of the CST complex (CTC1–STN1–TEN1), which plays an essential role in telomere replication and the protection of chromosomal ends. The CST complex functions in a manner similar to replication protein A (RPA), helping maintain telomere stability and preventing replication-associated DNA damage [37,38]. Pathogenic variants in *CTC1* are known to cause telomere biology disorders such as Coats plus syndrome [39] and cerebroretinal microangiopathy with calcifications and cysts (CRMCC) [40], both of which involve retinal vascular abnormalities and cerebral microangiopathy. Dyskeratosis congenita is another example of a telomere maintenance disorder resulting from impaired telomere stability [41]. The presence of vascular abnormalities across these conditions supports a potential role for *CTC1* in maintaining cerebral microvascular integrity.

Telomere shortening and dysfunction of the CST complex can contribute to endothelial senescence, increased oxidative stress, and impaired vascular repair [39,40]. Endothelial dysfunction has been repeatedly linked to migraine pathophysiology, particularly in migraine with aura, where altered vascular reactivity and impaired neurovascular coupling may promote cortical spreading depression (CSD) [20]. In addition, endothelial dysfunction [42] and oxidative stress [43] may contribute to trigeminovascular sensitisation and neuroinflammation. Subtle disruption of telomere maintenance could therefore affect small vessel integrity and increase migraine susceptibility through microvascular instability, impaired cerebral autoregulation, or increased vulnerability to CSD. The nominal significance of rs62624978 in the joint model (OR = 0.55, *p* = 0.011), together with its strong signal in the unadjusted PLINK analysis (OR = 0.217, *p* = 0.0014), supports the possibility of a protective effect of the variant allele on migraine risk, potentially through altered CST complex function. To our knowledge, no previous studies have reported an association between rs62624978, or *CTC1* more broadly, and migraine susceptibility. This suggests that the finding may represent a novel genetic signal linking telomere biology and migraine, although independent replication is needed.

### 4.4. Biological Interpretation: ASTN2 and Neuronal Circuitry

rs6478241 within *ASTN2* (NM_001365069.1; chr9:119252629) showed one of the strongest and most consistent association signals in this study. It remained significant after Bonferroni correction in the joint model (OR = 0.54, 95% CI 0.37–0.79, *p* = 0.002) and after FDR correction in the marginal model (OR = 0.57, 95% CI 0.41–0.77, q = 0.003). Previous studies also support the relevance of this variant. A Chinese case–control study including 581 migraine cases and 533 controls identified rs6478241 as a significant risk factor for migraine without aura and among individuals with a family history of migraine [44]. In addition, a large genome-wide association study (GWAS) involving 23,285 migraine cases and 95,425 controls reported heterogeneity in SNV effects across migraine subtypes, although the direction of effect for rs6478241 remained consistent [45]. Replication of this signal in our cohort, even after adjustment for covariates and multiple testing, strengthens the evidence supporting ASTN2 as a true migraine susceptibility locus.

ASTN2 (Astrotactin 2) encodes a neuronal adhesion molecule involved in glial-guided neuronal migration and the formation of cortical circuits [46]. Through its interactions with the extracellular matrix, ASTN2 contributes to neuronal positioning, connectivity, and synaptic organisation. Dysregulation of ASTN2 has been linked to neurodevelopmental disorders such as autism spectrum disorder [47], further supporting its importance in maintaining neuronal integrity [48]. Because migraine is associated with altered cortical excitability and increased network sensitivity, genetic variations in *ASTN2* may influence the stability of neural circuits involved in pain processing and sensory signalling. Changes in neuronal migration and connectivity may also interact with vascular dysfunction, potentially affecting neurovascular coupling and increasing susceptibility to cortical spreading depression (CSD) [49].

### 4.5. Biological Interpretation: rs7304841 (12p12) as a Risk-Increasing Variant

rs7304841, located in the 12p12 chromosomal region, was the only SNV in this study to show a risk-increasing association with migraine that reached FDR significance in the marginal model (OR = 1.51, 95% CI 1.13–2.01, *p* = 0.005, q = 0.035) and remained nominally significant in the joint model (OR = 1.61, 95% CI 1.11–2.35, *p* = 0.013). In contrast, this variant was not significant in the unadjusted PLINK analysis performed on the full cohort (*n* = 548; OR = 1.181, *p* = 0.31), suggesting that the association became clearer after adjustment for covariates and imputation of missing data. This may indicate confounding in the unadjusted analysis or that the true signal was better detected after correction for population stratification and other covariates. The missingness rate for rs7304841 was relatively low at 7.6%, indicating that the imputed estimates were largely supported by observed genotype data. The biological role of variants in the 12p12 region in migraine susceptibility is still unclear, and replication in an independent cohort will be important to determine whether this association is genuine.

### 4.6. Statistical Considerations

From a statistical standpoint, the consistency of signals across the PLINK unadjusted analysis, the MICE-imputed marginal models, and the MICE-imputed joint model increases confidence in the strongest findings. Adjustment for population structure using PC1–PC4 appeared effective, as PCA showed no clear clustering differences between cases and controls. In the joint model, L2-regularised logistic regression helped reduce the risk of overfitting given the inclusion of 21 predictors in a sample of 304 individuals, improving the stability of coefficient estimates. Pooling results across M = 20 imputed datasets using Rubin’s rules also ensured that uncertainty introduced by imputation was appropriately reflected in the final standard errors and *p*-values.

The joint model showed good discriminative performance, with an AUC of 0.822. Using a classification threshold of 0.50, the model correctly classified 219 of 304 participants, giving an overall accuracy of 72.0%, with sensitivity of 79.9% and specificity of 62.9%. The higher sensitivity compared with specificity suggests that the model was better at identifying migraine cases than correctly excluding controls, which is commonly seen in genetic risk models involving multiple modest-effect variants. However, this should not be interpreted as evidence of clinical predictive value. The observed AUC likely reflects separation within the current study sample and would require independent external validation before any prognostic use could be considered. The AUC reported here (0.822) is also slightly lower than the 0.832 reported in a previous analysis of the same dataset, likely reflecting differences in missing data handling, imputation strategy, and sample selection.

An important methodological aspect of this study was the formal assessment and treatment of missing genotype data. Eleven of the 21 SNVs had missingness greater than 20%, with a mean per-SNV missing rate of 30.3%. The MCAR test showed that missingness was associated with case–control status in 13 of 21 SNVs (*p* < 0.05), supporting a MAR rather than MCAR mechanism. Under MAR, complete-case analysis can lead to biased estimates, making MICE imputation with Rubin’s rules the more appropriate approach. The general directional agreement between the five available complete-case estimates and the MICE-imputed ORs (Supplementary Figure S2) provides some reassurance, although the complete-case sample consisted of only five participants and was far too small for meaningful quantitative comparison.

### 4.7. Limitations

Several limitations should be considered when interpreting these findings. First, the candidate gene approach, although biologically driven, restricts analysis to pre-selected loci and therefore limits the potential to identify novel associations. It may also introduce bias toward previously characterised pathways.

Second, the final analytic sample of 304 individuals after PCA-based merging is likely underpowered to detect the small effect sizes typically observed in complex traits such as migraine, particularly after correction for multiple testing. Post-hoc power analyses (Supplementary Tables S2 and S3) indicated that the study was adequately powered (≥80%) to detect moderate-to-large effects in common variants (MAF ≥ 0.20) at the full sample size (*n* = 548). However, power dropped substantially for rare and low-frequency variants (MAF < 0.05) across both sample sizes. In particular, for variants with MAF < 0.02, power remained below 30% even at an OR of 3.0 in the reduced dataset (*n* = 304), meaning that negative findings for these variants should be interpreted cautiously, as true effects may have been missed.

Third, the lack of an independent replication cohort limits the generalisability of the findings, and the results should therefore be viewed as exploratory and hypothesis-generating. While the ethnically homogeneous sample reduces the risk of population stratification, it also restricts how broadly the findings can be applied to other populations. In addition, the relatively high mean age of participants (~76 years) may introduce survivorship and recall bias, given that migraine is typically more prevalent earlier in life, which could influence both phenotype classification and allele frequency estimates.

Fourth, despite careful quality control procedures, the exclusion of variants deviating from Hardy-Weinberg equilibrium and LD pruning may have inadvertently removed variants of biological relevance. Stratified analyses by migraine subtype were also not performed due to limited statistical power.

Fifth, although missing data were addressed using the MICE approach, imputed genotypes for SNVs with >50% missingness (six SNVs in this dataset) remain associated with considerable uncertainty. As such, effect estimates for these variants should be interpreted with particular caution.

Finally, while several of the observed associations are biologically plausible, they do not establish causality. Functional validation studies are required to determine whether variants such as rs62624978 have a direct impact on CTC1 function or downstream neurovascular pathways.

Despite these limitations, the study has several strengths, including clinically confirmed ICHD-based phenotyping, high-quality genotyping, appropriate adjustment for population stratification, robust handling of missing data using MICE, and the use of complementary single-variant and joint modelling frameworks.

## 5. Conclusions

This study investigated the association between 21 biologically prioritised SNVs and migraine development in a case–control cohort of 304 individuals, using multiple imputation by chained equations (MICE) to address substantial genotype missingness (mean 30.3% per SNV) and applying multiple complementary logistic regression frameworks. Two variants, rs1052053 and rs6478241 in *ASTN2*, showed the most robust evidence, surviving Bonferroni correction in the joint multivariable model (*p* = 0.001 and *p* = 0.002, respectively) and also reaching significance after FDR correction in the marginal per-SNV analysis (q = 0.003 for both). A third variant, rs7304841 at 12p12, reached FDR significance in the marginal model (q = 0.035) and was nominally significant in the joint model (*p* = 0.013), suggesting a potential risk effect that requires replication. rs62624978 in *CTC1* showed its strongest association in the unadjusted PLINK analysis (OR = 0.217, *p* = 0.0014) and remained nominally significant in the joint imputed model (*p* = 0.011) but did not survive multiple-testing correction in any model.

The identification of rs6478241 in *ASTN2* at Bonferroni-level significance is consistent with prior reports from independent cohorts [36,37] and supports a role for neuronal adhesion and cortical circuit organisation in migraine susceptibility. The nominal association observed for rs62624978 in *CTC1*, a gene involved in telomere maintenance and previously implicated in cerebrovascular disease, extends the genetic architecture of migraine beyond classical ion channel mechanisms and supports a broader neurovascular model of disease. Overall, these findings are consistent with a shared genetic basis between migraine and cerebral small vessel disease, where pathways related to endothelial stability, telomere maintenance, and neurovascular coupling may jointly contribute to risk [29,30,31,32].

The joint model achieved an AUC of 0.822, indicating good discrimination between cases and controls beyond chance, although external validation is required before any clinical interpretation. Given the exploratory candidate-variant design, modest sample size, and absence of replication, all findings should be interpreted as hypothesis-generating.

Future work should focus on independent replication of rs1052053, rs6478241, rs7304841, and rs62624978 in larger and ethnically diverse cohorts. Fine-mapping and functional annotation studies will be required to identify causal variants within these loci. Integration of transcriptomic and epigenomic data from relevant neuronal and vascular tissues, particularly examining *CTC1* expression and CST complex function in endothelial cells, will be essential to clarify biological mechanisms. Expanding analyses to genome-wide approaches and incorporating migraine subtype stratification may further refine the genetic architecture of migraine.

## Figures and Tables

**Table 1:** PLINK v1.90 single-variant association analysis for 21 candidate SNVs with migraine (*n* = 548, unadjusted). Ordered by unadjusted *p*-value. Amber rows: nominal *p* < 0.05; red rows: *p* < 2.38 × 10^−3^ (Bonferroni). Bold *p*-values: *p* < 0.05. MAF, minor allele frequency obtained from the gnomAD database (non-Finnish European population); GC, genomic control; OR, odds ratio; Bonf, Bonferroni; FDR (BH), false discovery rate (Benjamini–Hochberg).

Chr	SNV	Gene	MAF	OR	*p* (unadj)	*p* (GC)	*p* (Bonf)	*p* (Holm)	Sidak-SS	Sidak-SD	FDR (BH)
17	rs62624978	*CTC1*	0.018	0.217	**0.001416**	**0.02175**	**0.02973**	**0.02973**	**0.02931**	**0.02931**	**0.02923**
9	rs6478241	*ASTN2*	0.630	0.345	**0.002783**	**0.03153**	0.05845	0.05567	0.05685	0.05422	**0.02923**
11	rs10895275	*YAP1*	0.376	0.703	**0.03876**	0.1373	0.814	0.7365	0.564	0.5282	0.2713
1	rs35737219	*MTHFR*	0.0149	2.353	0.1108	0.2515	1	1	0.915	0.8791	0.5711
1	rs1572668	*1p31.1*	0.443	0.790	0.136	0.2837	1	1	0.9536	0.9167	0.5711
1	rs1052053	*1q22*	0.370	0.696	0.1841	0.3395	1	1	0.986	0.9614	0.5815
10	rs140325655	*KCNK18*	0.009	0.63	0.2045	0.3617	1	1	0.9918	0.9677	0.5815
7	rs42039	*7q21*	0.271	0.793	0.2215	0.3794	1	1	0.9948	0.97	0.5815
12	rs1800127	*LRP1*	0.0199	0.785	0.2892	0.446	1	1	0.9992	0.9882	0.6311
12	rs7304841	*12p12*	0.404	1.181	0.3117	0.467	1	1	0.9996	0.9887	0.6311
13	rs34004222	*COL4A1*	0.005	1.816	0.3476	0.4995	1	1	0.9999	0.9909	0.6311
13	rs200735885	*COL4A2*	0.003	0.800	0.3606	0.511	1	1	0.9999	0.9909	0.6311
10	rs10786156	*PLCE1*	0.467	0.890	0.4338	0.5736	1	1	1	0.994	0.7007
16	rs8046696	*CFDP1*	0.561	0.792	0.4796	0.6112	1	1	1	0.9946	0.7193
17	rs62620189	*CTC1*	0.002	2.000	0.5686	0.6819	1	1	1	0.9972	0.7891
4	rs7684253	Near *REST/SPINK2*	0.561	0.936	0.6434	0.7392	1	1	1	0.9979	0.7891
12	rs1024905	Near *FGF6*	0.528	0.933	0.6521	0.7458	1	1	1	0.9979	0.7891
7	rs1008539	*ASB15/LMOD2/WASL*	0.441	1.058	0.6764	0.7641	1	1	1	0.9979	0.7891
14	rs11624776	*Near ITPK1*	0.349	1.057	0.7601	0.8262	1	1	1	0.9979	0.8401
1	rs880315	*CASZ1*	0.356	1.093	0.8548	0.8953	1	1	1	0.9979	0.8976
3	rs6790925	*TGFBR2*	0.387	0.991	0.97	0.9785	1	1	1	0.9979	0.97

## Data Availability

All data presented in this study are available on request from the corresponding author.

## Supplementary Materials

The following supporting information can be downloaded at: https://www.mdpi.com/article/10.3390/genes17050541/s1, Figure S1: Per-SNV Missingness, Figure S2: Sensitivity Analysis, and Figure S3: Missingness Heatmap; Table S1: missingness associated with Phenotype, Table S2: PLINK marginal analysis, and Table S3: MICE-imputed logistic models.
