# Supplementary material for: Exploring Vascular Contributions to Migraine: Association Analysis of Small Vessel Disease Genetic Variants
Source: Genes (Basel). 2026 May 1;17(5):541. doi: 10.3390/genes17050541 (PMC13205994; doi:10.3390/genes17050541)
Supplement: Supplementary file 1 [file genes-17-00541-s001.zip › genes-4220875-supplementary.pdf]

Supplementary Material

Supplementary Figure S1, Per-SNV Missingness

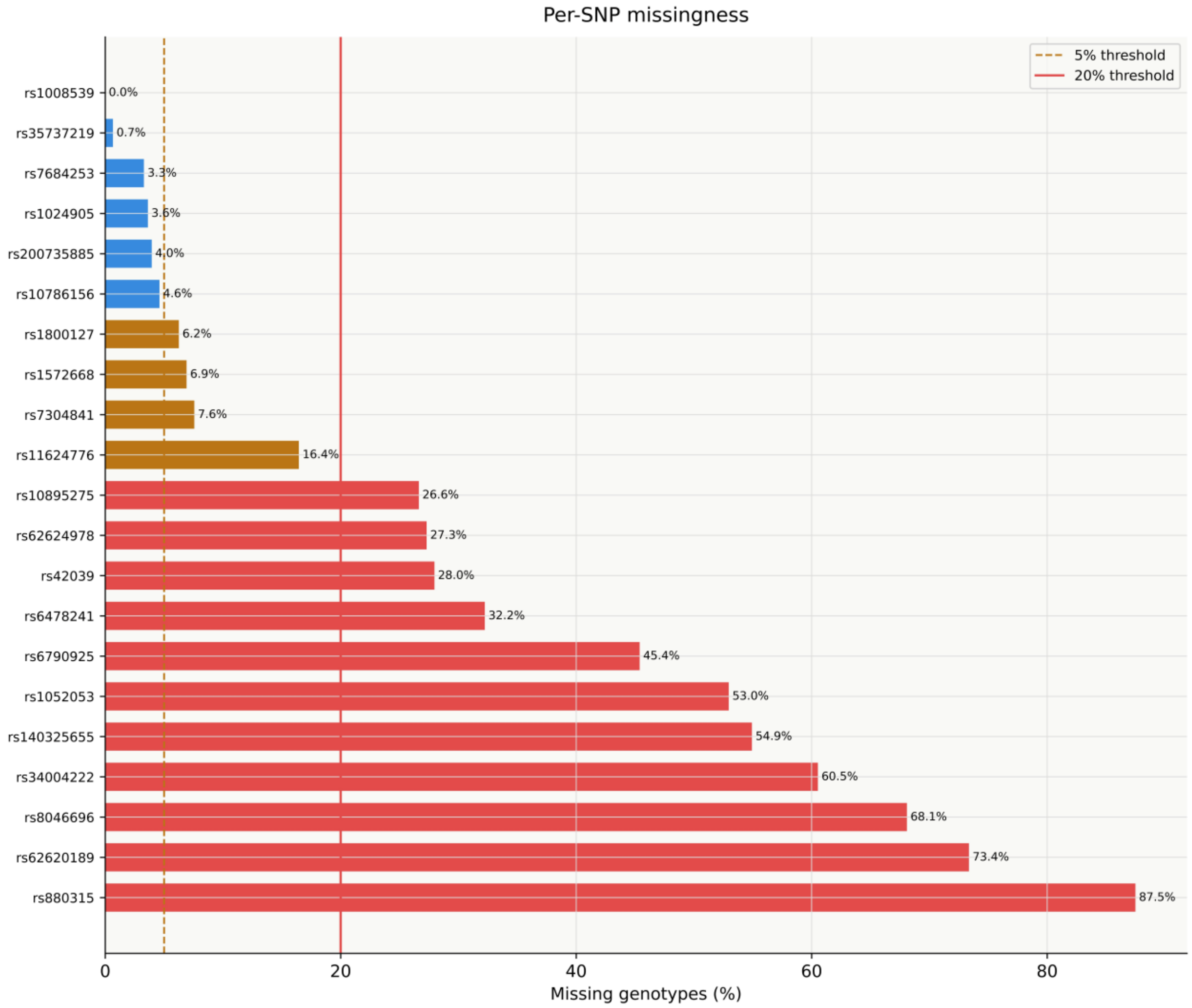

Supplementary Figure S1: Per-SNV genotype missingness rates across 21 candidate SNVs (N = 304). Ordered by ascending missingness. Amber dashed = 5% threshold; red solid = 20% threshold. Blue: < 5%; amber: 5–20%; red: > 20%. Range: 0.0% (rs1008539) to 87.5% (rs880315); mean 30.3%.

Supplementary Figure S2, Sensitivity Analysis

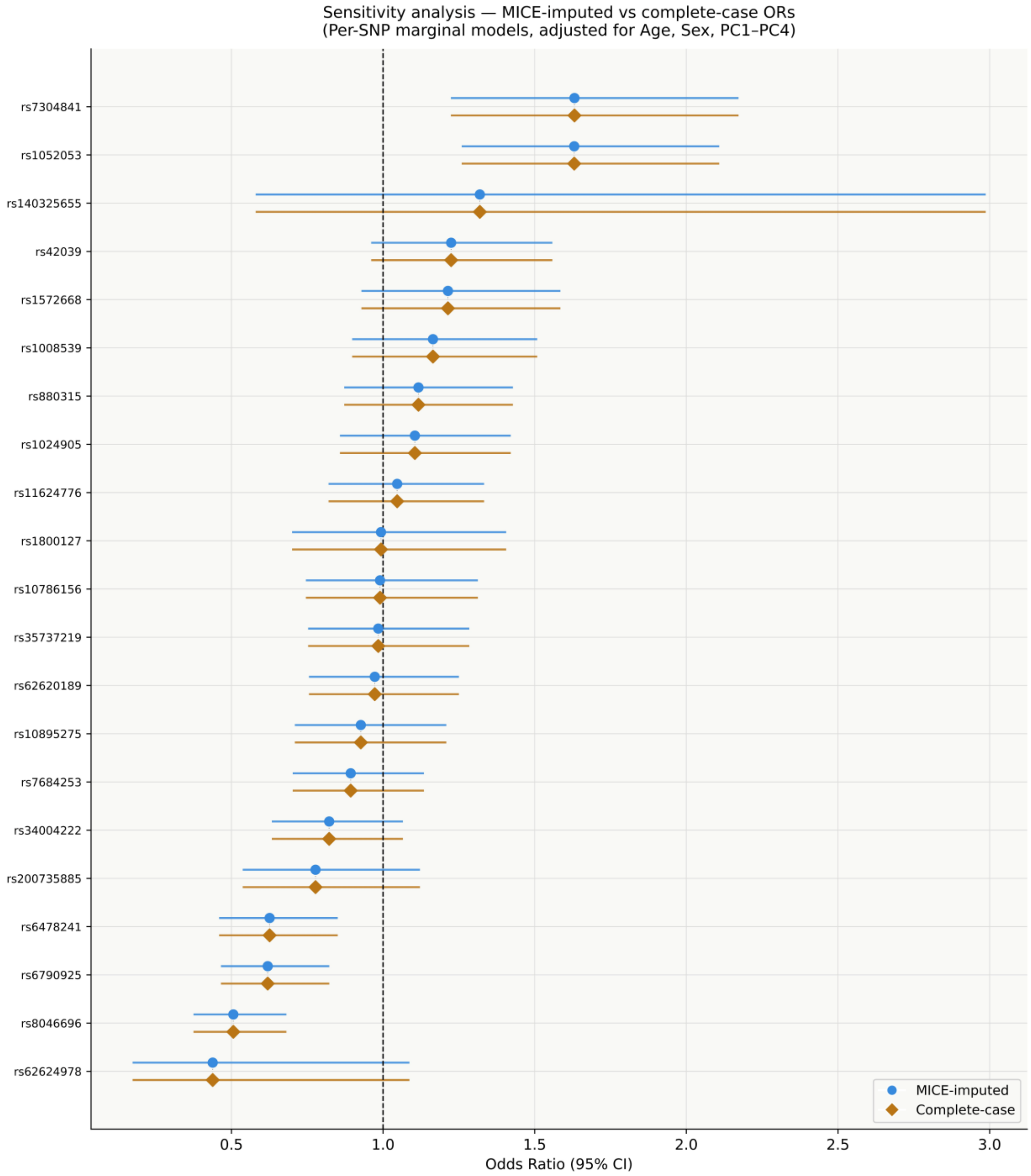

Supplementary Figure S2: Sensitivity analysis comparing MICE-imputed ORs (blue circles, N = 304) against complete-case ORs (amber diamonds, N = 5) for all 21 SNVs. Complete-case estimates are based on only 5 participants and are presented for directional comparison only. Dotted lines connect paired estimates for the same SNV.

Supplementary Figure S3, Missingness Heatmap

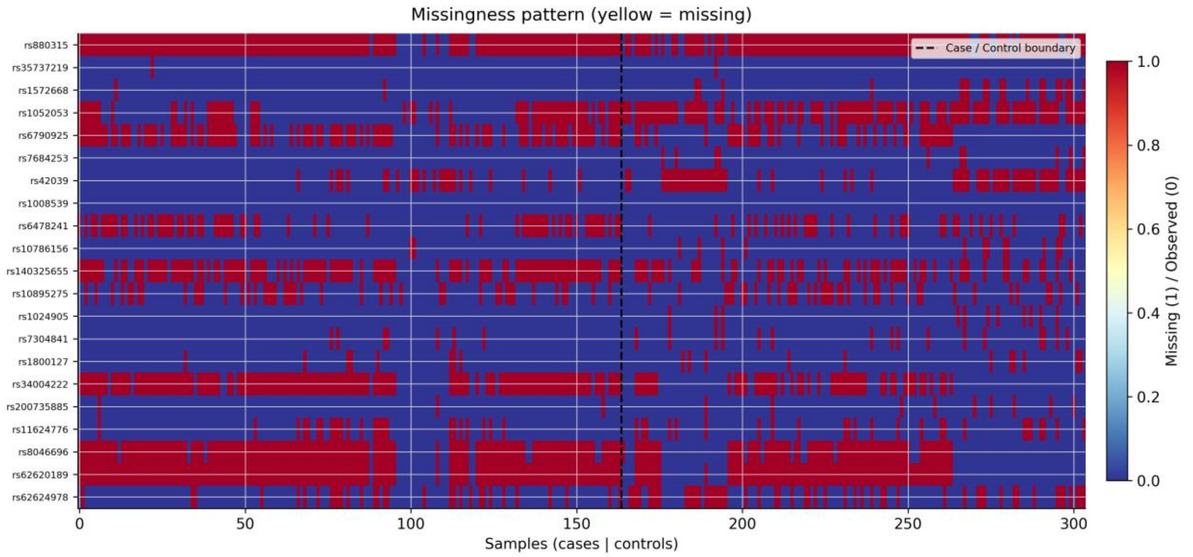

Supplementary Figure S3: Sample-level missingness pattern across 21 SNVs (N = 304). Rows = SNVs; columns = participants ordered cases (left) then controls (right of dashed line). Red = missing; blue = observed. Contiguous blocks of missingness are consistent with assay-level failure.

Supplementary Table S1, missingness associated with Phenotype

── MCAR test (missingness ~ Phenotype + covariates) ──────────

SNP	OR_missing	P_mcar	n_missing	MAR_flag
rs880315	0.7773	0.2384	266	False
rs35737219	NaN	NaN	2	False
rs1572668	0.3446	0.0014	21	True
rs1052053	0.4417	0.0000	161	True
rs6790925	1.9311	0.0000	138	True
rs7684253	0.2643	0.0144	10	True
rs42039	0.5472	0.0001	85	True
rs1008539	NaN	NaN	0	False
rs6478241	1.4239	0.0077	98	True
rs10786156	0.4392	0.0182	14	True
rs140325655	1.6984	0.0000	167	True
rs10895275	0.9433	0.6681	81	False
rs1024905	0.2438	0.0080	11	True
rs7304841	0.6034	0.0508	23	False
rs1800127	0.9079	0.7024	19	False
rs34004222	3.4139	0.0000	184	True
rs200735885	0.6197	0.1405	12	False
rs11624776	0.9022	0.5702	50	False
rs8046696	3.6383	0.0000	207	True
rs62620189	4.2930	0.0000	223	True
rs62624978	0.6437	0.0019	83	True

Supplementary Power Table S2

Table S2 | N = 548 (267 cases, 281 controls) PLINK marginal analysis

Applies to the unadjusted single-variant PLINK analysis on the full cohort.

MAF row	OR=1.5	OR=2	OR=2.5	OR=3	OR=4	OR=5	Study SNVs assigned to this row (gene, actual MAF)
**0.002**	5.6%	7.1%	9.1%	11.5%	16.9%	22.9%	rs62620189 (*CTC1*, MAF = 0.002) | rs200735885 (COL4A2, MAF = 0.003)
**0.005**	6.6%	10.4%	15.5%	21.6%	34.8%	48.0%	rs34004222 (*COL4A1*, MAF = 0.005)
**0.010**	8.2%	15.8%	26.1%	37.6%	59.6%	76.3%	rs140325655 (*KCNK18*, MAF = 0.009) | rs35737219 (MTHFR, MAF = 0.0149)
**0.020**	11.3%	26.5%	45.4%	63.2%	**86.6%**	**96.0%**	rs62624978 (*CTC1*, MAF = 0.018) | rs1800127 (LRP1, MAF = 0.0199)
0.050	20.4%	53.5%	**80.6%**	**93.8%**	**99.6%**	**100.0%**	—
0.100	33.7%	78.8%	**96.5%**	**99.6%**	**100.0%**	**100.0%**	—
**0.300**	61.3%	**97.4%**	**99.9%**	**100.0%**	**100.0%**	**100.0%**	rs42039 (7q21, MAF = 0.271) | rs11624776 (Near ITPK1, MAF = 0.349)
**0.400**	65.3%	**98.0%**	**100.0%**	**100.0%**	**100.0%**	**100.0%**	rs880315 (CASZ1, MAF = 0.356) | rs1052053 (1q22, MAF = 0.37) | rs10895275 (YAP1, MAF = 0.376) | rs6790925 (TGFBR2, MAF = 0.387) | rs7304841 (12p12, MAF = 0.404) | rs1008539 (ASB15/LMOD2/WASL, MAF = 0.441) | rs1572668 (1p31.1, MAF = 0.443)
**0.500**	65.2%	**97.7%**	**99.9%**	**100.0%**	**100.0%**	**100.0%**	rs10786156 (PLCE1, MAF = 0.467) | rs1024905 (Near FGF6, MAF = 0.528) | rs8046696 (CFDP1, MAF = 0.561) | rs7684253 (Near REST/SPINK2, MAF = 0.561)
**0.650**	58.5%	**94.8%**	**99.6%**	**100.0%**	**100.0%**	**100.0%**	rs6478241 (ASTN2, MAF = 0.63)

MAF = minor allele frequency; OR = odds ratio. Values are power (%) at α = 0.05 two-sided Wald test. ‡ Rows with no study SNVs shown for reference.

Supplementary Power Table S3

Table S3 | N = 304 (164 cases, 140 controls) MICE-imputed logistic models

Applies to per-SNV marginal and joint logistic regression models on MICE-imputed data with covariate adjustment.

MAF row	OR=1.5	OR=2	OR=2.5	OR=3	OR=4	OR=5	Study SNVs assigned to this row (gene, actual MAF)
**0.002**	5.3%	6.1%	7.2%	8.4%	11.1%	14.1%	rs62620189 (CTC1, MAF = 0.002) | rs200735885 (COL4A2, MAF = 0.003)
**0.005**	5.8%	7.8%	10.5%	13.5%	20.5%	27.9%	rs34004222 (COL4A1, MAF = 0.005)
**0.010**	6.7%	10.7%	16.0%	22.2%	35.5%	48.5%	rs140325655 (KCNK18, MAF = 0.009) | rs35737219 (MTHFR, MAF = 0.0149)
**0.020**	8.4%	16.4%	26.9%	38.4%	60.0%	76.2%	rs62624978 (CTC1, MAF = 0.018) | rs1800127 (LRP1, MAF = 0.0199)
0.050	13.2%	32.2%	53.9%	71.9%	**91.8%**	**98.0%**	—
0.100	20.5%	52.6%	78.8%	**92.3%**	**99.3%**	**99.9%**	—
**0.300**	38.4%	**82.2%**	**96.9%**	**99.6%**	**100.0%**	**100.0%**	rs42039 (7q21, MAF = 0.271) | rs11624776 (Near ITPK1, MAF = 0.349)
**0.400**	41.5%	**84.6%**	**97.5%**	**99.6%**	**100.0%**	**100.0%**	rs880315 (CASZ1, MAF = 0.356) | rs1052053 (1q22, MAF = 0.37) | rs10895275 (YAP1, MAF = 0.376) | rs6790925 (TGFBR2, MAF = 0.387) | rs7304841 (12p12, MAF = 0.404) | rs1008539 (ASB15/LMOD2/WASL, MAF = 0.441) | rs1572668 (1p31.1, MAF = 0.443)
**0.500**	41.6%	**83.8%**	**96.9%**	**99.5%**	**100.0%**	**100.0%**	rs10786156 (PLCE1, MAF = 0.467) | rs1024905 (Near FGF6, MAF = 0.528) | rs8046696 (CFDP1, MAF = 0.561) | rs7684253 (Near REST/SPINK2, MAF = 0.561)
**0.650**	36.8%	76.5%	**93.0%**	**98.0%**	**99.8%**	**100.0%**	rs6478241 (ASTN2, MAF = 0.63)

MAF = minor allele frequency; OR = odds ratio. The reduced N reflects PCA merging (samples with call rate < 50% excluded prior to PCA). Values are power (%) at α = 0.05 two-sided Wald test.
